# Influence of early neurological complications on clinical outcome following lung transplant

**DOI:** 10.1371/journal.pone.0174092

**Published:** 2017-03-16

**Authors:** Josep Gamez, Maria Salvado, Alejandro Martinez-de La Ossa, Maria Deu, Laura Romero, Antonio Roman, Judith Sacanell, Cesar Laborda, Isabel Rochera, Miriam Nadal, Francesc Carmona, Estevo Santamarina, Nuria Raguer, Merce Canela, Joan Solé

**Affiliations:** 1 Neurology Department, Vall d'Hebron University Hospital, Vall d'Hebron Research Institute, Autonomous University of Barcelona, Barcelona, Spain; 2 Department of Neurophysiology, Vall d'Hebron University Hospital, Autonomous University of Barcelona, Barcelona, Spain; 3 Department of Thoracic Surgery, Lung Transplant Unit, Vall d'Hebron University Hospital, Barcelona, Spain; 4 Department of Pulmonology, Lung Transplant Unit, Vall d'Hebron University Hospital, Barcelona, Spain; 5 Critical Care Department, Lung Transplant Unit, Vall d'Hebron University Hospital, Barcelona, Spain; 6 Department of Anesthesiology, Vall d'Hebron University Hospital, Barcelona, Spain; 7 Department of Statistics, University of Barcelona, Barcelona, Spain; National Yang-Ming University, TAIWAN

## Abstract

**Background:**

Neurological complications after lung transplantation are common. The full spectrum of neurological complications and their impact on clinical outcomes has not been extensively studied.

**Methods:**

We investigated the neurological incidence of complications, categorized according to whether they affected the central, peripheral or autonomic nervous systems, in a series of 109 patients undergoing lung transplantation at our center between January 1 2013 and December 31 2014.

**Results:**

Fifty-one patients (46.8%) presented at least one neurological complication. Critical illness polyneuropathy-myopathy (31 cases) and phrenic nerve injury (26 cases) were the two most prevalent complications. These two neuromuscular complications lengthened hospital stays by a median period of 35.5 and 32.5 days respectively. However, neurological complications did not affect patients’ survival.

**Conclusions:**

The real incidence of neurological complications among lung transplant recipients is probably underestimated. They usually appear in the first two months after surgery. Despite not affecting mortality, they do affect the mean length of hospital stay, and especially the time spent in the Intensive Care Unit. We found no risk factor for neurological complications except for long operating times, ischemic time and need for transfusion. It is necessary to develop programs for the prevention and early recognition of these complications, and the prevention of their precipitant and risk factors.

## Introduction

Lung transplantation has been established as a therapeutic option for patients in the terminal stages of pulmonary disease [1,2]. Approximately 3,500 people worldwide receive a transplant every year [3–7]. As a result of improved immunosuppression, more refined surgical techniques and precautions against post-transplant infections, the rates of survival for recipients of lung transplantation are 80–85% after one year and 50–55% after five years [6–9].

Neurological complications arise in around 72% of lung transplant recipients [9–15]. Post-transplant neurological complications after solid organ transplantation other than the lung are also common, with an incidence ranging between 10% and 59% depending on the series and the organ transplanted. For example, a third of patients undergoing liver transplants present neurological complications [16–17]. These complications are also common in renal transplant recipients, and contribute to morbidity and mortality [18]. Neurological complications occur following heart transplantation in 50–70% of patients, mostly during the perioperative period [19–20].

In lung recipients, serious complications involving the central nervous system (CNS) have been the focus of study to date, and neurological problems affecting the peripheral and autonomic nervous systems have until recently received little attention [21–23]. Although diseases of the peripheral nervous system (PNS) and the autonomic nervous system (ANS) have been reported post-lung transplant, the incidence of clinical characteristics and the impact of the neurological complications in the lung transplant population are generally unknown [13,24–27]. We report here on CNS, PNS and ANS complications in a large cohort of lung transplant recipients in a single institution in a two-year period.

## Methods

### Data collection

This is a retrospective cohort review reporting on the CNS, PNS and ANS concerns of 109 lung transplant recipients (66 men, 43 women) at a single institution over a two-year period (between January 2013 and December 2014). We subsequently reviewed the medical records, diagnostic imaging and electrophysiological investigations of all patients who underwent lung transplantation. The neurological complications were classified in three groups: those affecting the central nervous system (CNS), those affecting the peripheral nervous system (PNS), and those affecting the autonomic nervous system (ANS). Phrenic injury and critical illness myopathy-neuropathy were evaluated by electrophysiological studies. Gastrointestinal complications were defined as previously reported [20]. The neurological complications were codified according to the International Classification of Diseases (10th edition) [28]. The information was obtained after approval by our institutional review board (IRB).

### Statistical analysis

The data are presented as the mean, median, standard deviation, range and interquartile range for the continuous variables, and counts and percentages for the categorical variables. The data were compared between groups using the Student *t* test, the Mann–Whitney *U* test and the Brunner-Munzel test (heteroscedastic data), analysis of variance (ANOVA), analysis of covariance (ANCOVA) and the Kruskal-Wallis test by ranks, as appropriate for distribution normality. In the ANOVA and ANCOVA, log transformation was applied to the dependent variable to improve normality in some cases.

The proportions of patients presenting neurological complications and other categorical variables were compared using the chi-squared or Fisher’s exact test as appropriate. The association between the number of complications and the duration of surgery was studied with a Poisson regression model. Cox proportional hazard models were used to test the univariate associations between time-independent covariates, such as demographics, respiratory tests, ischemic time and post-transplant survival.

A Mantel-Haenszel test was used to determine the contribution of neurological complications to survival time. Survival was estimated using Kaplan-Meier methodology.

We performed a multivariate Cox regression model to study the dependency of survival time on predictor variables, including age at transplantation, uni- or bilateral transplant, ischemic times, total lung operating times, blood loss during surgery, use of ECMO and neurological complications.

Statistical analysis was performed using the R computing environment (R Core Team, 2014). A *p*-value of less than 0.05 was considered significant.

### Ethics statement

This retrospective study was approved by the Clinical Research Ethics Committee (CREC) of the Hospital Universitari Vall d’Hebron. None of the transplant donors were from a vulnerable population. Informed consent was obtained from all individual participants included in the study. Patient records/information was anonymized and de-identified prior to analysis. The study was conducted according to the principles set out in the Declaration of Helsinki.

## Results

### Patient characteristics

One hundred and nine patients underwent lung transplantation during the study period. One patient died within 24 hours of the transplant and was consequently excluded from the morbidity analysis. There were no cases of re-transplant in our series.

The main characteristics are summarized in Table 1. Forty-three (39.8%) of the 108 patients received single-lung transplantation via anterior thoracotomy through the fourth or fifth intercostal space. The transplant involved the left lung in 21 cases and the right lung in 22 cases. The remaining sixty-five (60.2%) patients received a double lung transplant performed by bilateral anterior thoracotomy and transverse sternotomy at the level of the fourth intercostal space. The mean total duration of the surgery was 288.3 ± 80.5 minutes (median 285.0 minutes; range 145–580 minutes).

**Table 1:** Characteristics of lung transplant recipients (n = 108).

Variable	N (%)	Neurological complications	No Neurological complications	P value	HR	95% CI	P value
**AGE AT TX**	47.6 ± 14.7	49.9 ± 12.3	45.6 ± 16.4	.317	1.01	(0.98,1.04)	.459
0–20	10 (9.3)	3	7				
21–40	13 (12.0)	3	10				
41–60	72 (66.7)	42	30				
> 60	13 (12.0)	3	10				
**GENDER**				.598			.978
MALE	66 (61.1)	33	33				
FEMALE	42 (38.9)	18	24		1.01	(0.46,2.23)	
**BMI**	24.4 ± 5.2	25.2 ± 4.8	23.6 ± 5.4	.218	0.98	(0.91,1.06)	.649
<30	93 (86.1)	45	48				
31–35	11 (10.2)	5	6				
>35	2 (1.9)	1	1				
**SPIROMETRY**							
FVC vol.	2.0 ± 0.7	2.0 ± 0.8	2.0 ± 0.7	.959	0.60	(0.32,1.13)	.097
FVC%	47.1 ± 15.7	46.0 ± 16.4	48.2 ± 15.0	.361	0.97	(0.94,1.00)	.047
FEV1 vol.	1.3 ± 0.7	1.3 ± 0.8	1.3 ± 0.6	.869	0.70	(0.38,1.30)	.246
FEV1%	39.1 ± 19.4	39.0 ± 21.0	39.3 ± 17.9	.547	0.98	(0.96,1.01)	.162
FV1%/FVC	63.8 ± 23.2	64.2 ± 23.3	63.5 ± 23.4	.931	1.00	(0.98,1.02)	.948
**TYPE TX**				.751			.723
BILATERAL	65 (60.2)	32	33				
UNILATERAL	43 (39.8)	19	24		1.15	(0.53,2.51)	
**HOSPITAL STAY**							
ICU	32.0 ± 35.6	49.5 ± 40.7	16.4 ± 20.5	< .001^(*)^	1.01	(1.00,1.02)	.003
WARD	18.0 ± 12.6	18.0 ± 13.6	17.9 ± 11.7	.863	0.88	(0.83,0.93)	< .001
TOTAL	50.0 ± 34.0	67.6 ± 37.4	34.3 ± 20.6	< .001^(*)^	1.01	(1.00,1.02)	.077
**PERIOPERATIVE PERIOD**							
ISCHEMIC TIME	312.5 ± 92.6	328.9 ± 76.2	297.6 ± 103.8	.023 ^(*)^	1.00	(0.99,1.01)	.715
<2 h	3 (2.8)	0	3				
2–4 h	17 (15.7)	5	12				
4–6 h	66 (61.1)	35	31				
>6 h	21 (19.4)	11	10				
TRANSFUSION				.005			.943
yes	80 (74.1)	45	35		0.97	(0.41,2.31)	
no	27 (25.0)	6	21				
TRANSFUSION number of pRBC				1.00	1.05	(0.98,1.12)	.265
<4	60 (75.0)	32	28				
4–8	11 (13.8)	6	5				
>8	7 (8.8)	4	3				
ECMO				.311			.212
yes	24 (22.2)	14	10		1.74	(0.76,4.01)	
no	82 (75.9)	36	46				
ECMO time (min)	188.5 ± 77.4	191.1 ± 69.4	184.0 ± 94.9	.859 ^(*)^	0.99	(0.98,1.01)	.316
< 120 min	5 (20.8)	3	2				
> 120 min	17 (70.8)	11	6				
**Operating time (min)**	288.3 ± 80.5	310.3 ± 76.1	267.7 ± 79.6	0.010^(*)^	1.00	(0.99,1.01)	.417
**TX INDICATION**				.903			.977
COPD	36 (33.3)	18	18				
IPF	36 (33.3)	18	18		0.84	(0.33,2.13)	
PHTN	8 (7.4)	3	5		0.93	(0.20,4.24)	
CF	11 (10.2)	4	7		0.62	(0.14,2.85)	
OTHER	17 (15.7)	8	9		0.95	(0.30,3.02)	

(*) Brunner-Munzel Test.

**Abbreviations**: BMI = Body Mass Index; CF = Cystic Fibrosis; CI = confidence interval; COPD = Chronic Obstructive Pulmonary Disease; ECMO = Extra-Corporeal Membrane Oxygenation; HR = :heart rate; IPF = Idiopathic Pulmonary Fibrosis; PHTN = Pulmonary Hypertension; pRBC = packed red blood cells.

Twenty-seven patients (24.8%) died during the follow-up period. The mean survival time among the patients who died was 177.6 days, with a median of 127 days and a range between 1 and 717 days. As the third quartile is 307.8 days, the data shows that 81.5% of those who died did so within one year. The median of the total hospital stay for the 108 patients was 38 days, with a median stay in the Intensive Care Unit (ICU) of 15.5 days.

### Distribution of neurological complications and survival analysis

We identified neurological complications in 51 (47.2%) of the 108 patients. These neurological complications are summarized in Table 2. Sixteen patients had one neurological complication, 9 had two neurological complications, 9 had three neurological complications, and 17 had four or more neurological complications (68.6% of the patients had 2 or more complications). The neurological complications were classified in three groups: those affecting the CNS, those involving the PNS and those affecting the ANS. Two PNS disorders–critical illness polyneuropathy/myopathy (31 cases) and phrenic nerve injury (26 cases)—were the most prevalent neurological complications in our group of lung TX patients. Psychomotor agitation (n = 8), cerebrovascular accident (stroke) (n = 5), encephalopathy (n = 2), and seizures (n = 2) were the most common neurological complications involving the CNS, and the most common ANS complication was gastroparesis, observed in 22 patients.

**Table 2:** Distribution of neurological complications.

CENTRAL NERVOUS SYSTEM			
	ICD-10	NUMBER OF OBSERVATIONS	MEDIAN APPEARANCE AFTER TX (days)
CVA (STROKE)	I64	5	16.0
SEIZURES	G40	2	23.5
ENCEPHALOPATHY	G93.4	2	31.0
PSYCHOMOTOR AGITATION	F05.8	8	27.5
TREMOR	R25.1	2	46.5
OTHER CNS COMPLICATIONS	G96	1	24.0
PERIPHERAL NERVOUS SYSTEM			
CIP/CIM	G72	32	35.0
PNI	G58.8	26	2.0
POLYNEUROPATHY	G62	1	8.0
RADICULOPATHY	M54.1	2	3.0
OTHER PNS COMPLICATIONS	G64	1	59.0
AUTONOMIC NERVOUS SYSTEM			
GASTROPARESIS	K31	19	8.0
MYDRIASIS	H57.0	1	31.0
OTHER ANS COMPLICATIONS	G90.8	3	31.0

**Abbreviations**: ANS = autonomic nervous system; CIP/CIM = critical illness polyneuropathy or myopathy; CVA = cerebrovascular accident (stroke); PNI = phrenic nerve injury; PNS = peripheral nervous system.

**Fig 1:**
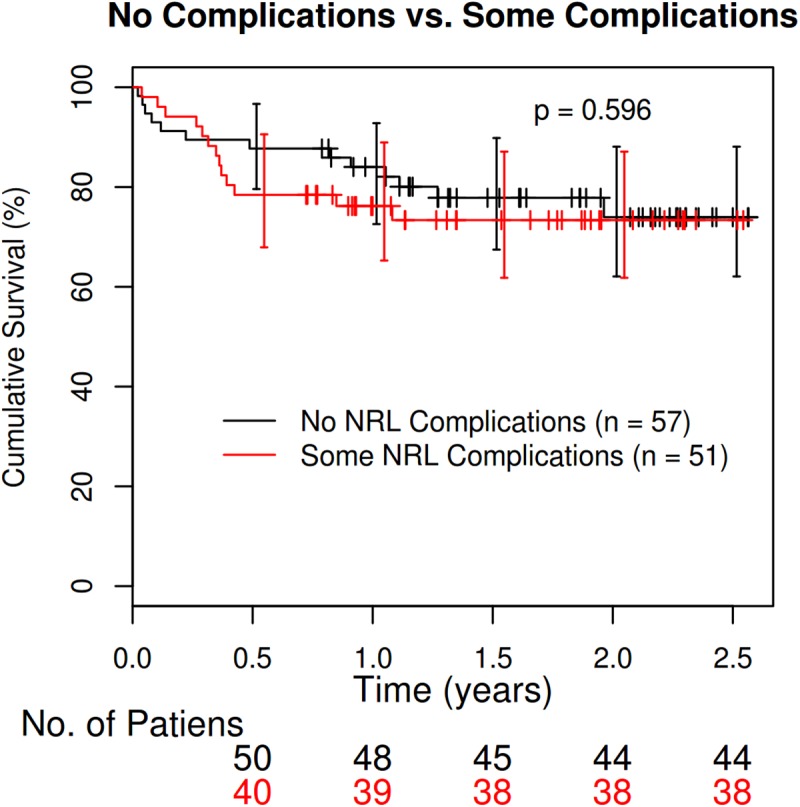
Effect of neurological complications on the Kaplan-Meier survival curve.

When we studied the estimated rate of survival for the 108 patients, we observed no significant differences in terms of the effect of the variables sex, age, type of transplant and type of pulmonary disease. The unadjusted survival rates were 90.83% at 3 months and 79.82% at 1 year. No differences were found between complicated and non-complicated rates (94.12% at 3 months and 76.47% at 1 year for complicated, 87.93% at 3 months and 82.76% at 1 year for uncomplicated) (Fig 1).

Analysis of the effects of neurological complications on mortality (n = 26) showed that the mean survival time in the group without any complications was 229.08 days, while among those who had neurological complications, the survival time was 139.77 days. Although the mean number of days until death is higher among those with no complications, the difference is not significant, as there is a high level of variability in the death-time variable (*p*-value 0.21 using the Welch *t*-test). When we compared survival times between the group with complications and the group without any neurological complications, we found no dependency on any of variables mentioned above. Survival was not dependent on the type of neurological complication (CNS, PNS or ANS).

### Risk factors for neurological complications

When we investigated the existence of a correlation between all the variables and the risk of a neurological complication, the patients at greatest risk of presenting neurological complications were those who had spent the most time in the operating theater, those requiring blood transfusion (although this was not related to the number of pRBC required) and those with longer ischemic times. The mean duration of surgery for cases with complications was 310.30 ± 76.1 minutes (median 315; range 180–580). The mean duration of surgery for cases with no complications was 267.66 ± 79.6 minutes (median 261.5; range 145–425). (Wilcoxon test W = 819.5; *p-*value = 0.01035). Although age was not observed to be a risk factor for neurological complications, elderly patients were predisposed to multiple (4 or more) neurological complications (F = 3.117, *p-*value = 0.0348). Analysis of neurological complications by group, according to whether they affect the CNS, PNS or ANS, also failed to identify risk factors.

### Characteristics and distribution of neurological complications

**Fig 2:**
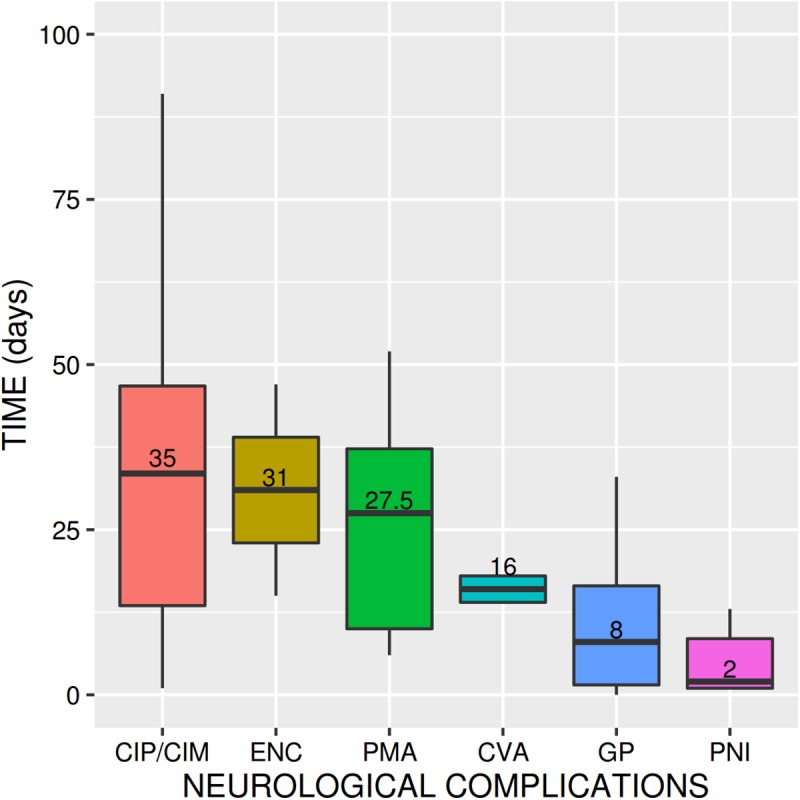
Post-transplant latencies (days) until appearance of neurological complications.

Most neurological complications appeared in the first two months (90.3%) post-transplant (see Fig 2). Early neurological complications include phrenic nerve injury (with a mean post-transplant latency of 9.7 ± 18.7 days), gastroparesis (13.1 ± 15.0 days), and seizures (23.5 ± 0.7 days), psychomotor agitation (13.1 ± 15.0 days), and stroke (30.2 ± 32.9 days). Critical illness polyneuropathy/myopathy (48.5 ± 66.4 days) and tremor (46.5 ± 4.9 days) occur later.

When classified in groups, neurological complications affecting the PNS sometimes appear within 30 days of transplant (39 versus 23) with a *p*-value = 0.024 for the Wilcoxon rank test for true location at 30 days or less. Twelve cases of neurological complications affecting the CNS occurred in the first month (versus 8 cases after 30 days; *p*-value = 0.32 for the Wilcoxon rank test). There are also significant differences between the ANS and appearance more than 30 days post-transplant (15 occurring within 30 days versus 6 occurring after 30 days, *p*-value = 0.0008574 for the Wilcoxon rank test).

### Hospital stays of lung recipients with neurological complications

The length of hospital stay (on the ward, in the ICU and total) was calculated for patients with and without complications. The average length of time spent in hospital (for these 108 patients) was 50.01 ± 34.0 days (median 38; range 8–167), with a mean of 32.04 ± 35.6 days (median 15.5; range 1–147) in the ICU, and 17.97 ± 12.6 days (median 16, range 0–65) in the Thoracic Surgery Unit (ward) (see Table 3).

**Table 3:** Comparative length of hospital stays.

	Total Cohort (n = 108)			Neurological complications (n = 51)			No neurological complications (n = 57)			*Non-parametric test*
	Mean (SD)	Median	Range	Mean (SD)	Median	Range	Mean (SD)	Median	Range	*p*-value
**ICU**	32.04 (±35.6)	15.5	1–147	49.53 (±40.7)	42	2–147	16.39 (±20.5)	8	1–97	8.768e-11
**WARD**	17.97 (±12.6)	16	0–65	18.04 (±13.6)	16	0–65	17.91 (±11.7)	16	0–58	0.863
**TOTAL**	50.01 (±34.0)	38.0	8–167	67.57 (±37.4)	56	14–167	34.3 (±20.6)	29	8–108	4.161e-13

**Fig 3:**
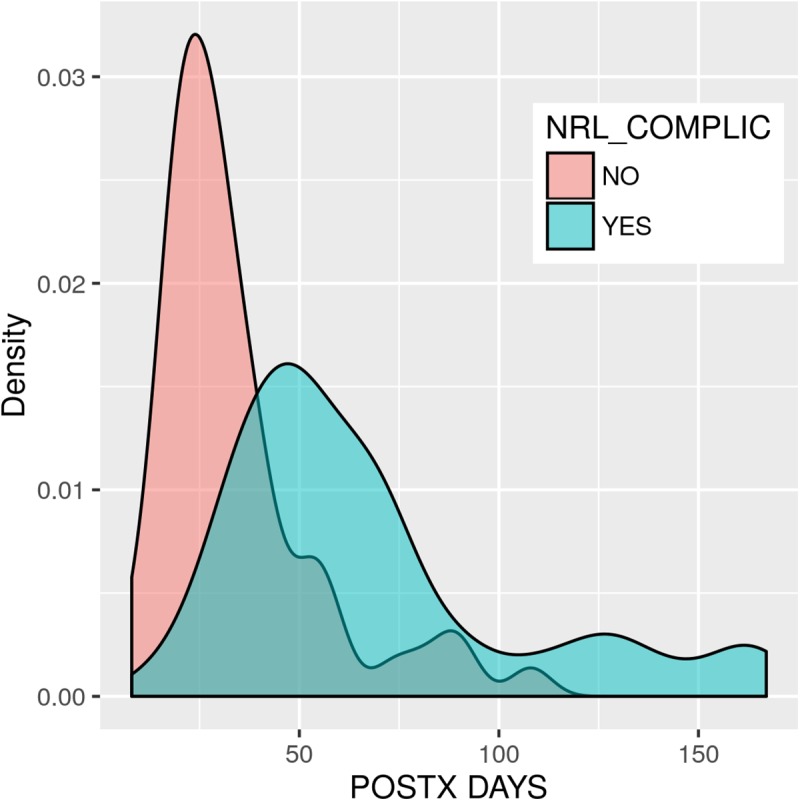
Effect of neurological complications on length of hospital stay.

The presence of neurological complications was a risk factor for a longer total hospital stay and a longer stay in the ICU. The average total length of hospital stay among the group without any complications was 34.3 ± 20.6 days, versus 67.6 ± 37.4 days for the group with neurological complications (Fig 3). The median for those without complications was 29 days, and for those with complications it was 56 days (*p-*value = 3.072e-09 by Wilcoxon test). This longer period for patients with neurological complications was due to a longer stay in the ICU. The average total time spent in the ICU in the group without any complications was 16.4 ± 20.5 days, versus 49.5 ± 40.7 days for the group with neurological complications. The median for those without complications was 8 days, and for those with complications it was 42 days (*p-*value = 3.392e-08 by the Wilcoxon test). No statistically significant differences for the stay on the thoracic surgery ward were found in either group (*p-*value = 0.863).

An analysis of the total time spent in hospital among the group with neurological complications shows a correlation between the mean length of hospital stay and the number of complications, with the group with four or more complications admitted for the longest period of time. Logarithmic transmission corrects the heteroscedasticity (*p*-value = 0.01647, Levene’s test) of the dependent variable total hospital admission time. The ANOVA test between the logarithm of the total time spent in hospital and the number of neurological complications is thus significant (*p-*value = 0.000205). In short, there are significant differences depending on the number and type of neurological complications experienced by the patient, which lengthen the time spent in hospital.

### Univariate and multivariate analysis

The univariate analysis performed includes gender, age at transplantation, type of transplant (uni- or bipulmonary), BMI, ischemic time, total time of operation, blood loss during surgery, use of ECMO, and all categories of neurological complication. Except for long operating times, ischemic times and requirement for transfusion (but not the number of pRBC required), none of the variables had a significant effect on the analysis.

The following variables were observed to be independent prognostic factors for neurological complications: ischemic time (p = .023), with an HR of 1.00 and a 95% CI of 0.99 and 1.02, the need for blood transfusion (p = .005), with an HR of 0.97 and a 95% CI of 0.41 and 2.31, and longer operating times (p = .01), with an HR of 1.00 and a 95% CI of 0.99 and 1.01. Neither age, gender, BMI spirometry, type of transplant (uni- or bilateral), ECMO nor the type of pulmonary disease leading to the transplant were independent prognostic factors for neurological complications.

In the multivariate Cox regression model to study the dependency of survival time, taking sex, age, type of pulmonary disease, time spent in the ICU and time on the ward as regression variables, only the variable “time on the ward” showed a significant association with survival time with Pr(> І z l) of 8.04e-05 and Pr(>ChiSq) 5.759e-06 in the partial likelihood ratio test in Cox model terms.

## Discussion

Despite improvements in protocols for the screening, management and medical-surgical treatment of recipients of lung transplantation, survival rates at 5 years do not exceed 60%. Although a better selection of patients, the search for risk factors influencing the outcome, improvement in pre-, per-, and postoperative techniques and the standardization of protocols for procedures in both the operating theater and in the ICU have been proposed, the rates of morbidity remain very high. There is evidence that long-term survival is directly related to postoperative complications. Some of these complications, such as rejection and infections, have been studied extensively [9, 29–30]. Interestingly, postoperative neurological complications in the outcome of lung transplants, and especially as a risk factor for a long stay in hospital, have not been studied in depth. This study aims to investigate the incidence of early neurological complications affecting the CNS, PNS and ANP in a series of 108 lung transplants, and whether their presence correlates with morbidity and prolonged hospital stays.

The incidence of patients with complications (51/108) is consistent with those described in other retrospective series [11–13]. Recipients may present more than one neurological complication, with 68% of patients presenting two or more neurological complications in a two-month period immediately after transplant. Despite the high incidence of neurological complications, they do not affect mortality. The rate of survival 12 months post-transplant is 81.5%, a similar proportion to the figure reported at highly specialized lung transplantation centers.

Previous studies have analyzed the frequency of neurological complications, mainly focusing on the CNS or on isolated neurological complications [9,12,13,22,23,25]. This explains why the rate of neurological complications in lung transplantation recipients ranges between 11.5% and 66% [12,13,22,23,25]. To the best of our knowledge, this is the first time that the entire spectrum of neurological complications after lung transplantation (i.e. those affecting the CNS, PNS and ANS) has been investigated. However, it is not known whether other factors apart from those mentioned could influence the inter-series variability of the frequencies observed, and the main bias factor is probably that all the studies are retrospective. Most neurological complications occur in the first two months post-transplant, have an impact on the length of hospital stay, and on the length of stay in the ICU in particular. The difference in the length of stay in the ICU between patients presenting complications and those who do not– 28.4 days–has a high financial cost and affects the recovery of lung transplant recipients. When we investigated risk factors for neurological complications, we found no relationship between the variables studied and possible neurological complications [13], including previous state before surgery and drugs administered. Age at transplant, gender, and type of transplant (uni- or bipulmonary) have been proposed as risk factors in previous series. We found that among our patients with complications, older individuals presented the most complications, and patients who had spent the most time in the operating theater were more likely to have complications, although these differences were not statistically significant.

Critical illness polyneuropathy/myopathy (CIP/CIM) was the most prevalent complication (29.6%) in our study, with a median post-transplant latency of 35 days. To the best of our knowledge, this neuromuscular complication has previously been described in only two studies. In the Pennsylvania series, only 4 of the 90 lung transplant recipients presented CIP/CIM [13]. In the Minnesota series, of the 95 lung transplant patients who had at least one neurological complication, 3 presented CIP/CIM [12]. Although the true incidence of CIP/CIM in lung transplant recipients is unknown, it is very frequent among patients receiving mechanical ventilation, transplant recipients, and those with severe pulmonary disorders, sepsis and burns [31–36]. Some reviews suggest that it may occur in 46% [34–36]. Based on these reviews, our findings and the average length of hospital stay after lung transplantation, the real incidence of CIP/CIM in lung recipients is probably underestimated. Early identification of this complication seems wise in view of the impact of CIP/CIM on patients [37]. First, their weaning period is significantly longer than in those without CIP/CIM, with a consequent longer stay in the ICU and an increased likelihood of respiratory infections. Second, the muscle wasting and weakness present in many of these patients with CIP/CIM leads to a significant functional limitation when they are discharged from the ICU.

Phrenic nerve injury (PNI) was the second most frequently observed neurological complication in our series (23.8%). PNI, a potentially important cause of respiratory failure, was first reported in 1963 in a group of four patients who underwent aortic valve replacement [37]. It also occurs after lung transplantation and is generally secondary to phrenic nerve injury during surgery. Its true incidence is unknown, and it was prospectively evaluated in patients with isolated lung transplantation for the first time in 1995, with an incidence of 29.6% [25]. Subsequent studies have found an incidence ranging from 2% to 90% [5,11,12,26,38–41]. Bilateral transplantation and male gender were found to be risk factors for PNI in some series, although not in our series. PNI has been considered the fourth most important complication of post-lung transplant respiratory failure, after pulmonary edema, acute allograft rejection and pulmonary sepsis. This complication usually appears in the first few days post-surgery, and lengthens the post-operative stay in hospital by an average of one month. Another result of PNI is a lengthened UCI stay of 9.28 days (geometric mean). Intra-operative electrophysiological phrenic nerve monitoring is a recommended measure for preventing damage to the phrenic nerve [42–46].

Encephalopathy, cerebrovascular accident (stroke), and seizures, in that order, were the most frequent CNS complications. Our findings are similar to those reported in other adult series. For example, in 2010 Mateen et al. found that the most common neurological complications in the CNS were strokes (12 patients), encephalopathy (29 patients), and seizures (7 patients) [12]. Strokes, and especially the hemorrhagic forms, are risk factors for mortality. Seizures are the most common CNS complication in pediatric populations, occurring in 27% of cases [11].

Neurogastroenterological complications were found in 24 patients. Gastroparesis was the most common, in 22 cases. Gastroparesis following lung transplants—considered a neuropathic disorder which alters the movement of food in the gastrointestinal tract—is common, with an incidence of up to 95% of cases [24,27,47]. It has been attributed to preexisting lung disease, intra-operative damage (inadvertent vagotomy or vagal injury during dissection of the posterior mediastinum) and medications. For many authors, it is a surgical complication, especially when it appears during the first days post-transplant. Gastroparesis is particularly important, as it affects the absorption of medication, delays correct oral nutrition and is a risk factor for bronchoaspiration.

The weaknesses of our study are those common to most retrospective studies. They include risk of bias, especially due to misclassification or selection, inter-rater variability, lack of consistency in control over record keeping, and failure to measure other possible risk factors.

In short, neurological complications are common among lung transplant recipients and their real incidence is very likely to have been underestimated. They usually appear in the first two months after surgery. Although they do not affect mortality, they do affect the mean length of hospital stay, and above all the time spent in the ICU. A significant number of patients present more than three neurological complications. We found no risk factor for neurological complications except for long operating times, ischemic times and requirement for transfusion (but not the number of pRBC required). This study provides an analysis of a full spectrum (CNS, PNS and ANS) of neurological complications after lung transplantation. Consensus guidelines and recommendations are necessary to define the requirements for prevention and early detection of neurological complications in patients scheduled to receive a lung transplant, including intra-operative electrophysiological phrenic nerve monitoring and the presence of a neurologist in multidisciplinary lung transplantation teams [45,46].

## Abbreviations

ANS: autonomic nervous system
CIP/CIM: critical illness polyneuropathy/myopathy
CF: cystic fibrosis
CNS: central nervous system
COPD: chronic obstructive pulmonary disease
CVA: cerebrovascular accident (stroke)
EMG: electromyography/electrophysiological studies
ENC: encephalopathy
GP: gastroparesis
PNI: phrenic nerve injury
ICU: intensive care unit
IPF: idiopathic pulmonary fibrosis
PHTN: pulmonary hypertension
PMA: psychomotor agitation
PNS: peripheral nervous system
SZ: seizure
TX: transplant
